# Modeling of Optimized Lattice Mismatch by Carbon-Dioxide Laser Annealing on (In, Ga) Co-Doped ZnO Multi-Deposition Thin Films Introducing Designed Bottom Layers

**DOI:** 10.3390/nano13010045

**Published:** 2022-12-22

**Authors:** Jaeyong Yun, Min-Sung Bae, Jin Su Baek, Tae Wan Kim, Sung-Jin Kim, Jung-Hyuk Koh

**Affiliations:** 1 School of Electrical and Electronic Engineering, Chung-Ang University, Seoul 06974, Republic of Korea; 2 Department of Intelligent Energy and Industry, Chung-Ang University, Heukseok-ro, Seoul 06974, Republic of Korea; 3 College of Electrical and Computer Engineering, Chungbuk National University, Cheongju 28644, Republic of Korea

**Keywords:** TCO, (In, Ga) co-doped ZnO, lattice mismatch, FoM, CO_2_ laser annealing

## Abstract

In this study, modeling of optimized lattice mismatch by carbon-dioxide annealing on (In, Ga) co-doped ZnO multi-deposition thin films was investigated with crystallography and optical analysis. (In, Ga) co-doped ZnO multi-deposition thin films with various types of bottom layers were fabricated on sapphire substrates by solution synthesis, the spin coating process, and carbon-dioxide laser irradiation with post annealing. (In, Ga) co-doped ZnO multi-deposition thin films with Ga-doped ZnO as the bottom layer showed the lowest mismatch ratio between the substrate and the bottom layer of the film. The carbon-dioxide laser annealing process can improve electrical properties by reducing lattice mismatch. After applying the carbon-dioxide laser annealing process to the (In, Ga) co-doped ZnO multi-deposition thin films with Ga-doped ZnO as the bottom layer, an optimized sheet resistance of 34.5 kΩ/sq and a high transparency rate of nearly 90% in the visible light wavelength region were obtained.

## 1. Introduction

Transparent conducting oxides (TCOs) are important materials for optoelectronic applications, such as light-emitting diodes (LEDs) [1,2], flat panels [3], and solar cells [4,5], due to their high electrical conductivity and optical transparency. Currently, indium–tin-oxide (ITO) is one of the most widely used TCO materials for next-generation transparent devices. However, some drawbacks need to be addressed concerning the use of TCOs for their related applications. Indium is a metal that is toxic, so it can pose a threat to the environment and humans. In addition, owing to its scarcity, it is also very expensive. Therefore, many studies have been conducted to find alternative materials that can overcome these limitations. Among these alternatives, zinc oxide is a reasonable alternative material due to its advantages of cost-effectiveness, non-toxicity, and high transparency. However, pure ZnO exhibits poor electrical properties [6]. Aiming to enhance the properties, several dopants have been considered for zinc oxide materials, such as In, Al, and Ga [7,8,9]. Among them, indium has the potential to improve the electrical properties such as sheet resistance or resistivity [10]. Moreover, gallium appears to be a stable dopant material, which is more efficient in preventing unexpected chemical reactions than other dopant materials [11]. When both of these elements are doped into ZnO, they can substitute Zn^2+^ in the ZnO wurtzite structure and exist as 3^+^ or 4^+^ ions. The substituted ions can provide excessive free electrons by oxygen vacancies in the ZnO structure. Particularly, with even small amounts of dopants, the electrical properties of ZnO can be significantly improved. Therefore, by introducing the co-dopants of (In, Ga), we may expect a lower sheet resistance combined with a higher chemical stability of TCO materials. The optical annealing produced by the laser can be used as an alternative to the conventional annealing method and can even enhance the electrical properties or induce surface heating. Furthermore, optical annealing heat treatment such as the rapid thermal process, direct optical irradiation, and the laser process in the UV–IR range can significantly improve the stress relief and electrical conductivity of ZnO-based thin films. [12,13,14]. These optical annealing processes have the advantages of shorter annealing times and lower annealing temperature conditions compared with other processes. In particular, a type of laser annealing process, carbon-dioxide laser annealing with a wavelength of 10.6 μm is a notable post annealing process with its excellent properties of of deep penetration depth, micron-meter scale processing, and energy controllability [15,16,17].

When the films are deposited onto the substrates, a difference in the lattice parameters between the substrates and thin films can occur. The stress or strain from the lattice mismatch is an undesirable factor in the thin film process, which is caused by the difference in lattice parameters between the substrate and the thin film [18,19]. This stress or strain in the thin film can interfere with the optical and electrical conductivity. Thus, through the annealing processes, we can expect that the characteristic of multi-deposition thin films can be enhanced by relieving the stress between the substrates and the thin films.

Until now, ZnO-based thin films have been fabricated using different types of deposition techniques such as pulsed laser deposition (PLD) [20,21], atomic layer deposition (ALD) [22,23], atmospheric pressure chemical vapor deposition (APCVD) [24,25], sol-gel spin coating [26,27], and magnetron sputtering [28]. In this research, the solution synthesis and spin coating process were introduced to deposit (In, Ga) co-doped multi-deposition thin films on sapphire substrates owing to the advantages of low cost, large-area deposition, and controllability of stoichiometry and film thickness [29]. In the first step of this study, we investigated the lattice mismatch between the substrates and various types of (In, Ga) co-doped multi-deposition thin films. We then analyzed the effects of carbon-dioxide laser annealing on each thin film with a different lattice mismatch in relation to each substrate.

## 2. Materials and Methods

Four types of multi-deposition (In, Ga) co-doped ZnO (IGZO) thin films were manufactured by solution synthesis and the spin coating method on sapphire substrates by varying the bottom layer of the film. As bottom layers, In-doped ZnO (IZO), Ga-doped ZnO (GZO), (In, Ga) co-doped ZnO (IGZO), and Ti doped ZnO (TZO) were prepared as the bottom layer of the multilayer thin film. The precursor was used as zinc acetate dehydrate (Zn(CH_3_COO)_2_.2·H_2_O) and the solvent and stabilizer were manufactured as 2-methoxyethanol and mono-ethanolamine (MEA), respectively. As dopant materials, indium nitrate hydrate (In(NO_3_)_3_ · H_2_O), gallium nitrate hydrate (Ga(NO_3_)_3_ · H_2_O), and titanium(IV) butoxide (Ti(C_4_H_9_)_4_) were applied. The molar ratio of zinc acetate dihydrate to mono-ethanolamine was set at 1.0. The composition ratios of In, Ga, and Ti were (1.5 mol%:0 mol%:0 mol%), (0 mol%:1.5 mol%:0 mol%), (1.0 mol%:1.0 mol%:0 mol%), (0 mol%:0 mol%:1.5 mol%) for IZO, GZO, IGZO, and TZO for the bottom layer materials, respectively. Furthermore, the composition ratio of In:Ga was (1.0 mol%:1.0 mol%) for the (In, Ga) multi-deposition thin films. The solutions were stirred at 60 °C for 24 h. Then, they were deposited onto sapphire substrates and spin-coated at 3000 rpm for 30 s. After the deposition, to evaporate the solvents and to eliminate the unnecessary elements and organic compounds, the multiple-layer thin films were cured in a room atmosphere at 300 °C for 10 min on a hotplate. Next, thermal annealing in an electrical furnace at 650 °C for 1 h was conducted for crystallization. After the electrical furnace process, the CW carbon-dioxide laser annealing process (Continuous wave CO_2_ laser, Autowin/Hardram, Ansan, Republic of Korea; output power: 1.5 Watt; frequency: 5 kHz; process time: 2.5 s) was applied to reduce the stress and the defects and to improve the crystallinity and the electrical properties. The crystallography measurements such as crystal structures and lattice parameters of the films were investigated by X-ray diffraction (XRD, D8-Advance/Bruker-AXS, Karlsruhe, Germany).

(1) $$d=\frac{n\lambda }{2sin\theta }$$

(2) $$\frac{1}{d_{hkl}^{2}}=\frac{4}{3}\left(\frac{h^{2}+hk+k^{2}}{a^{2}}\right)+\frac{l^{2}}{c^{2}}=\frac{l^{2}}{c^{2}}$$

(3) $$\frac{c}{a}=1.6028-13.4\times 10^{-7}T-2.7\times 10^{-9}T^{2}$$

The interplanar spacing was calculated using Bragg’s law (Equation (1)), where n is an integer (1, 2, 3, 4 ⋯), λ (0.154 nm) is the X-ray wavelength of the CuK_α_ source, and θ is the Bragg diffraction angle [30]. The lattice parameter c is calculated using Equation (2) where d_hkl_ is the interplanar spacing of the (hkl) index and a and c are the lattice parameters [31]. Equation (3) represents the ratio of the lattice parameters a to c according to the measurement temperature, where T is the absolute temperature [32,33]. The surface morphology of the films was investigated by field emission scanning electron microscopy (FE-SEM, SIGMA 300, Carl Zeiss, Jena, Germany). The 4-point probe method was conducted for the electrical properties, that is, the sheet resistance. We performed optical analysis by UV-vis spectrometry and photoluminescence spectra. Photoluminescence (PL, UniSAM-300/Uninanotech, Yongin, Republic of Korea) spectra were obtained using a spectrophotometer for bandgap energy with a He–Ag (200–250 nm) laser at room temperature. For electrical characterization, a four-point probe method (Sheet resistance measurement, Ossila, Rotherham, United Kingdom; probe spacing: 1.27 mm; target current: 0.12 μA; maximum voltage: 10 V; voltage increment: 0.1 V) was introduced. For optimization modeling, we compared and analyzed the crystallographic, optical, and electrical properties according to the introduction of four types of bottom layers namely IZO, IGZO, IGZO, and TZO as the bottom layers, respectively. Based on results, we confirmed the relationship between the material properties and the lattice mismatch ratio.

## 3. Results and Discussion

**Figure 1:**
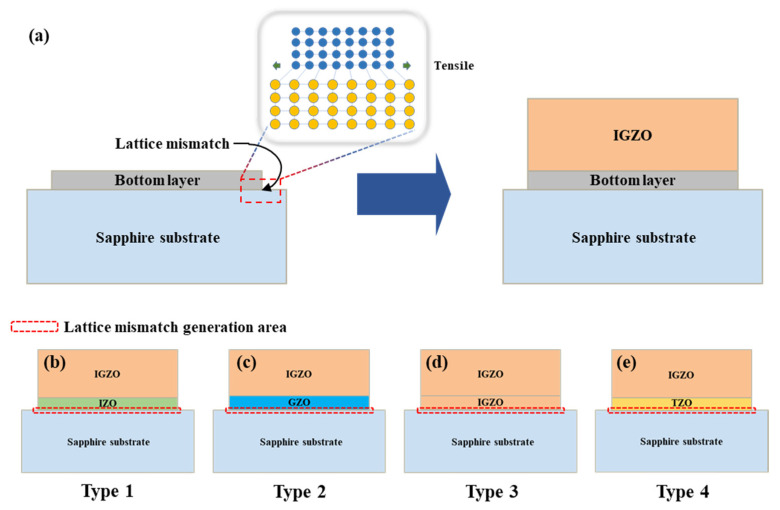
The overview of thin film structure (**a**) and the schematic diagram of 4 types of (In, Ga) co-doped ZnO multilayered thin film structures: Type 1 (**b**), Type 2 (**c**), Type 3 (**d**), Type 4 (**e**).

Figure 1 displays an overview of the thin film structure and a schematic diagram of the four types of (In, Ga) co-doped ZnO multi-deposition thin films. As shown in Figure 1a, lattice mismatch occurs owing to the difference in lattice parameters between the substrate and the bottom layer. The four different types of thin films have in common the top six layers of (In, Ga) co-doped ZnO multi-layer thin films with varying bottom layers: with In-doped ZnO (IZO) as the bottom layer for type 1 (Figure 1b), Ga doped ZnO (GZO) as the bottom layer for type 2 (Figure 1c), (In, Ga) co-doped ZnO (IGZO) as the bottom layer for type 3 (Figure 1d), and Ti doped ZnO (TZO) as the bottom layer for type 4 (Figure 1e). Each type represents a structure with a different lattice mismatch between the substrate and the bottom layer and all the films had a thickness of about 450 nm.

**Figure 2:**
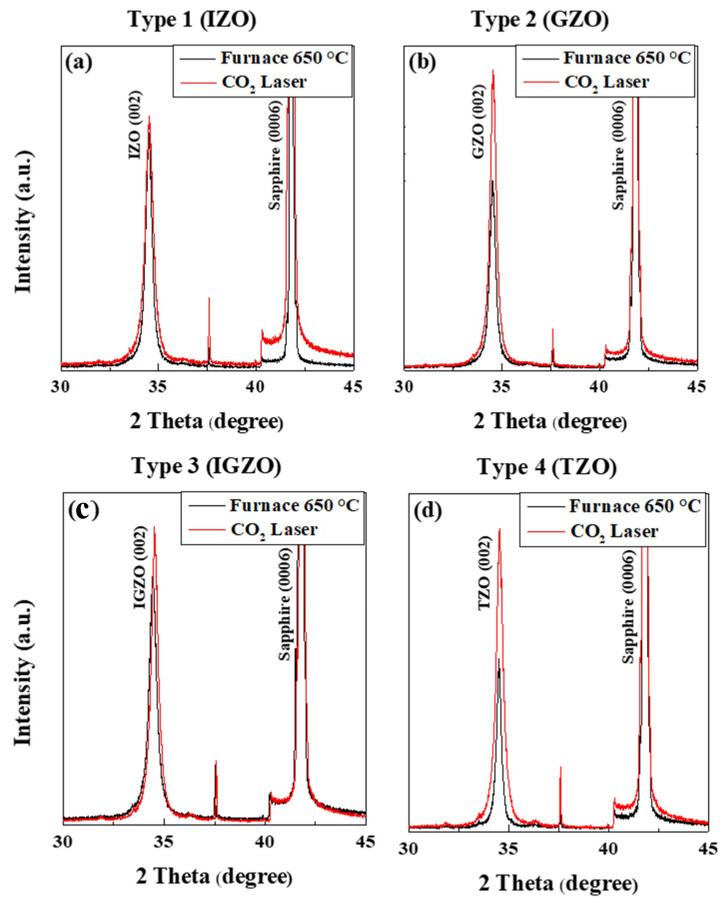
X-ray diffraction patterns of ZnO-based thin films after electrical furnace (**a**) and furnace & CO_2_ laser annealing in four type; (**a**) Type 1: IZO, (**b**) Type 2: GZO, (**c**) Type 3 (IGZO), (**d**) Type 4 (TZO).; Scan speed: 3.0 deg/min; measuring range: 30–40 degrees; furnace: 650 °C; CO_2_ laser: 1.5 W, 2.5 s.

Figure 2 illustrates the X-ray diffraction (XRD) patterns of the ZnO-based thin films, which were deposited as the bottom layers of the four types of (In, Ga) co-doped ZnO samples. All four patterns showed that the (002) peak positions were slightly shifted to lower angles after the carbon-dioxide laser annealing process. All the measurements were performed at room temperature (T = 298 K). By using Equations (2) and (3), we can derive each film’s lattice parameter a. Table 1 shows the (002) peak angles, lattice parameter c, and lattice parameter a for the ZnO based thin films subjected to different annealing processes; electrical furnace annealing (F) was performed, followed by sequential carbon-dioxide laser annealing (FL). As presented in Table 1, the Ga doped ZnO thin film showed the highest value of lattice parameter a, followed by (In, Ga) co-doped ZnO thin films, In doped ZnO thin films, and Ti doped ZnO thin films for both two different annealing processes. After carbon-dioxide laser annealing, the XRD peak intensity of the (In, Ga) co-doped ZnO multi-deposition thin films increased as depicted in Figure 2c. This implies that the carbon-dioxide laser post annealing process improved the crystallinity of the thin films. Hence, we established that all types of (In, Ga) co-doped ZnO thin films were well deposited and crystallized on the sapphire substrates.

**Figure 3:**
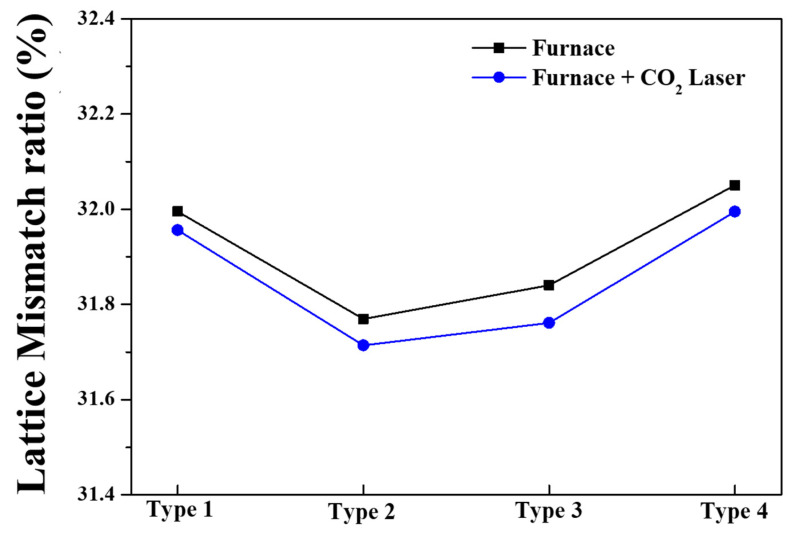
Lattice mismatch ratio of 4 types of (In, Ga) co-doped multilayered thin films after electrical furnace and furnace & CO_2_ laser annealing.

Figure 3 depicts the lattice mismatch ratio of the four different types of (In, Ga) co-doped multi-deposition thin films against the sapphire substrate. The mismatch ratio was calculated by the following Equation [33]:

(4) $$Mismatch\;ratio=\frac{a_{sapphire}-a_{film\;}}{a_{sapphire}}\times 100\;\left(\%\right)$$

where a_sapphire_ and a_film_ are lattice parameter a of sapphire substrate (a = 4.754 Å) [34] and bottom layer of (In, Ga) co-doped multi-deposition thin film, respectively.

After the electrical furnace process, type 2 exhibits the lowest mismatch ratio between the substrate and the film. It can be observed that types 2, 3, 1, and 4 have the lowest mismatch ratio in that order. After the carbon-dioxide laser annealing process was applied, the lattice mismatch ratio of all the (In, Ga) co-doped multi-deposition thin films diminished. This was caused by a rise in the lattice parameter a of the thin film, which was calculated from the XRD peaks shown in Figure 2 and presented in Table 1. Even when carbon-dioxide laser annealing was applied, there was no change in the order of the lattice mismatch ratio of the thin films. The calculated mismatch ratio was 31.95%, 31.71%, 31.76%, and 31.99% for type 1, type 2, type 3, and type 4 in the (In, Ga) co-doped ZnO multi-deposition thin films, respectively. The misfit strain can be expressed as follows [35]:

(5) $$\varepsilon =\frac{a_{substrate}-a_{film}}{a_{substrate}}$$

and is caused by the lattice mismatch ratio. According to Vlassak [18], for a thin film prepared on a sapphire substrate with a grain size L_0_ and the grain size increasing to L after the annealing in the electrical furnace, the volumetric strain compared to the initial state is as follows:

(6) $$\Delta V^{XS}=3\Delta a\left(\frac{1}{L}-\frac{1}{L_{0}}\right)$$

where Δa is the excess volume per unit of the grain boundary. The misfit strain and volumetric strain have the following correlation:

(7) $$\varepsilon =-\frac{1}{3}\Delta V^{XS}=\Delta a\left(\frac{1}{L}-\frac{1}{L_{0}}\right)$$

and stress:

(8) $$\sigma =M\Delta a\left(\frac{1}{L_{0}}-\frac{1}{L}\right)=M\varepsilon$$

where M is the biaxial modulus of the thin film. Thus, through the above-mentioned equations, it can be seen that lattice mismatch, misfit strain, and stress are in a directly proportional relationship with each other. Therefore, the stress was reduced owing to a decrease in the lattice mismatch due to carbon-dioxide laser annealing.

**Figure 4:**
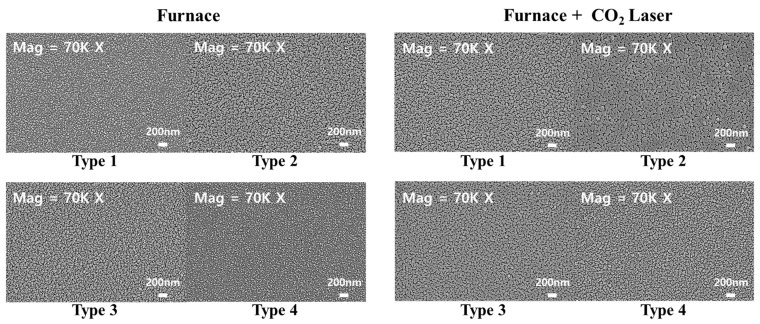
FE-SEM surface images of 4 types of (In, Ga) co-doped ZnO multilayered thin films after electrical furnace and furnace & CO_2_ laser annealing. Acceleration voltage: 5.00 kV; magnification: 70.00 K X; working distance: 4.8 mm. Image resolution pixel: 1024 × 768 pixel.

Figure 4 shows the FE-SEM surface morphology of (In, Ga) co-doped multi-deposition thin films with electrical furnace, electrical furnace plus carbon-dioxide laser annealing analyzed by field emission scanning electron microscopy (FE-SEM). As shown in Figure 4, type 2 exhibited the largest crystallite size for both the electrical furnace and the furnace & carbon-dioxide laser annealing processes. The crystallite size decreased in the order of type 3, type 1, and type 4. By comparing the images of the two different annealing processes, we can see that the crystallite size was slightly increased after carbon-dioxide laser annealing was applied. This result is caused by the high-energy irradiation of the carbon-dioxide laser annealing process. The crystallites of the (In, Ga) co-doped ZnO multi-deposition thin films absorbed the energy of the laser irradiation process and the crystallite size became larger than that of the films for which only the electrical furnace was processed. The calculated crystallite sizes of the (In, Ga) co-doped multi-deposition thin films are arranged in Table 2. Table 2 indicates the crystallite sizes of the four types of (In, Ga) co-doped multi-deposition thin films deposited on sapphire substrates. The crystallite sizes were calculated by the Scherrer equation [36,37]:

(9) $$D=\frac{K\times \lambda }{B\times cos\theta_{B}}$$

where λ is the X-ray wavelength (0.154 nm) of the CuKα source, K is an appointed constant of 0.89, θ_B_ is the constant of Bragg’s angle, and B is the full width at the half maximum (FWHM) values of the (002) diffraction peaks in the XRD data presented in Figure 3. The calculated crystallite size values coincided with the FE-SEM images. Moreover, the grain growth values after carbon-dioxide laser annealing were 0.66, 1.37, 0.77, and 0.61 nm for type 1, type 2, type 3, and type 4, respectively. Here, the role of the energy of the carbon-dioxide laser is the driving force for grain growth in the thin films [38]. When the carbon-dioxide laser is irradiated on a thin film, energy is used to relieve the stress of the thin film. The remaining energy assists in grain growth. Grain growth tends to decrease, where high stress or strain remains in the structure. Figure 4. shows that the lattice mismatch was reduced through carbon-dioxide laser annealing, indicating that carbon-dioxide laser annealing relieved the stress in the thin film. Thus, we can derive the relieved stress data from the reduced lattice mismatch ratio. Because the carbon-dioxide laser energy is used to relieve the stress, the remaining energy of the carbon-dioxide laser can be estimated from the relieved stress data. Additionally, grain growth tends to decrease in total energy stored in the thin films, such as strain energy and dislocation energy. These energies are produced by the existence of defects or dislocations [19]. Considering the residual stress in the thin film and the remaining carbon-dioxide laser energy, type 2 is the most suitable state for grain growth. Therefore, the calculated lattice mismatch in the film can be the main factor in increasing the grain size of the structure. The optical annealing energy from the carbon-dioxide laser was first applied to the lattice-mismatched area to cure the mismatch instead of the grain growth process. As a result, we believe that the lower lattice-mismatched type 2 has a lower resistance with higher grain growth values, while the higher lattice-mismatched type 4 has higher resistance with lower grain growth values.

**Figure 5:**
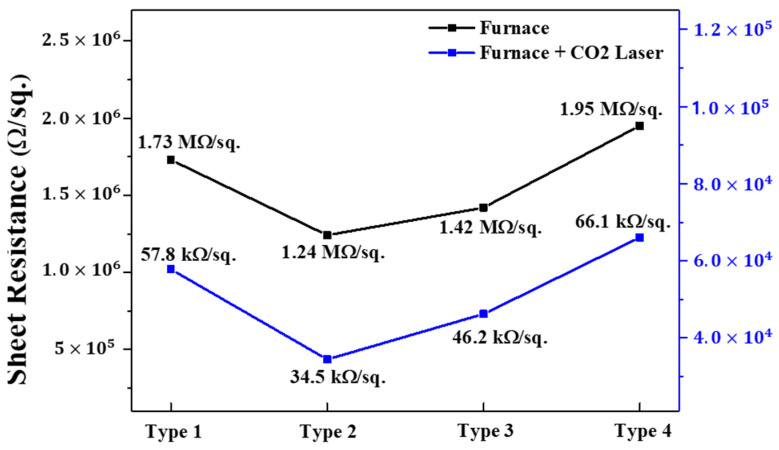
Sheet resistance of 4 types of (In, Ga) co-doped ZnO multilayered thin films after electrical furnace and furnace & CO_2_ laser annealing.

**Figure 6:**
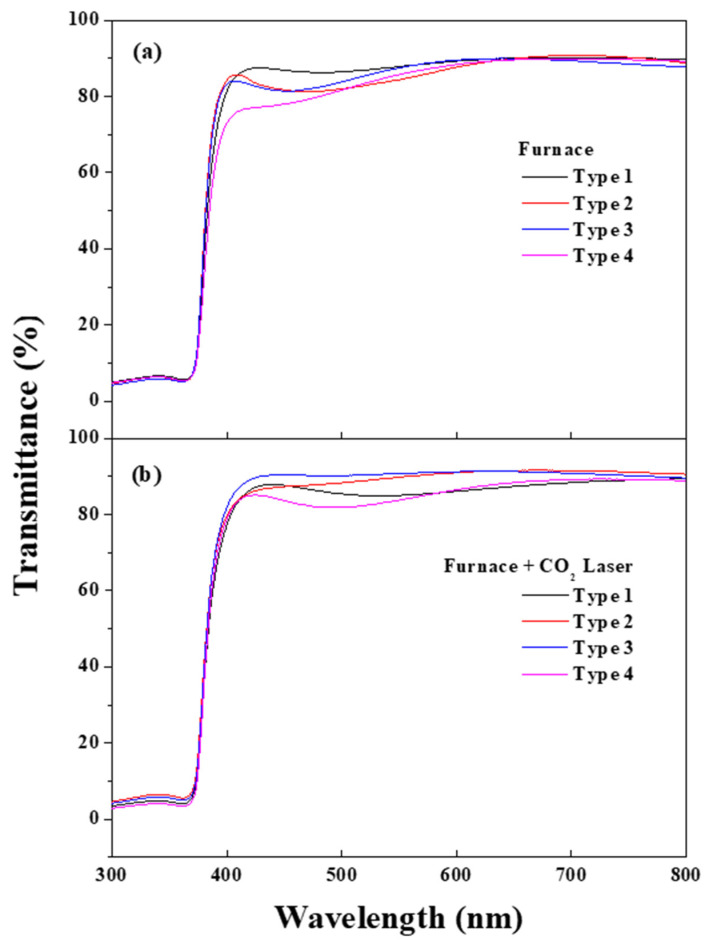
Optical transmittance of 4 types of (In, Ga) co-doped multilayered thin films after electrical furnace (**a**) and furnace & CO_2_ laser annealing (**b**).

Figure 5 illustrates the sheet resistance of four different types of (In, Ga) co-doped ZnO multi-deposition thin films measured following electrical furnace and after furnace and carbon-dioxide laser annealing processes sequentially. When measured after the electrical furnace process, the type 2 specimen depicted the lowest sheet resistance value of 1.24 MΩ/sq., while the other types exhibited higher sheet resistances than type 2, and all types of (In, Ga) co-doped multi-deposition thin films had MΩ/sq. level of sheet resistance. When the carbon-dioxide laser annealing was applied following the electrical furnace process on the thin films, the sheet resistance drastically decreased. In order to exist as a substitutional form of (In, Ga) at the Zn site, more than the formation energy of substitutional (In, Ga) should be applied [39]. (In, Ga) were well substituted with Zn through the energy of the carbon-dioxide laser, which generated free electrons and increased the conductivity of the thin films. Moreover, the carbon-dioxide laser annealing process can remove defects in the thin film and induce grain growth. The electrons, which are charge carriers of (In, Ga) co-doped multi-deposition thin films, are trapped in defects or scattered in grain boundaries [40]. These phenomena degrade the conductance of the thin films. However, by introducing carbon-dioxide laser irradiation, high optical energy can be transmitted to the thin film, and the stress can be relieved. As a result, grain growth can be induced. Therefore, carbon-dioxide laser annealing leads to a reduction in defects and grain boundary scattering. As depicted in Figure 6, the sheet resistance decreased from MΩ/sq. to kΩ/sq. level after the carbon-dioxide laser annealing process. Type 2 depicted the lowest sheet resistance of 34.5 kΩ/sq.

**Figure 7:**
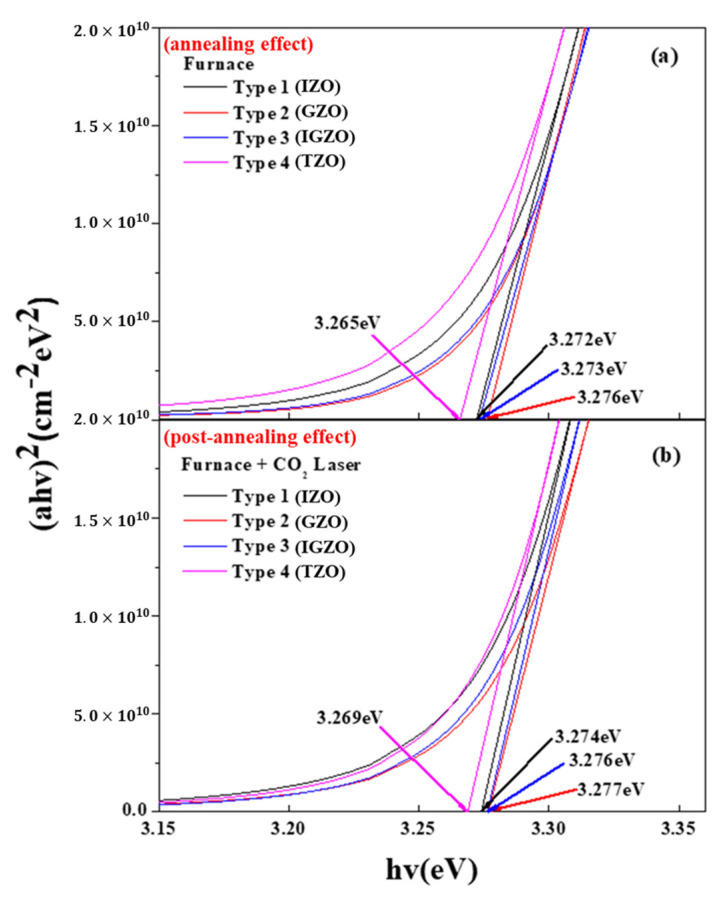
Energy band gap of 4 types of (In, Ga) co-doped multilayered thin films after electrical furnace-annealing effect (**a**) and furnace & CO_2_ laser annealing–post-annealing effect (**b**).

Figure 6 displays the optical transmittance measurement for four different types of (In, Ga) co-doped multi-deposition thin films following the electrical furnace (a) and after the furnace and carbon-dioxide laser sequential irradiation process (b). As depicted in Figure 7a,b, the average optical transmittance data of all types at wavelengths from 400 to 800 nm, which is the wavelength range of the visible region, were higher than 85%. The average optical transmittance data indicate that all four different types of (In, Ga) co-doped multi-deposition ZnO thin films have high optical transmittance properties in the visible region and are suitable for optical device applications such as transparent conducting oxides.

**Figure 8:**
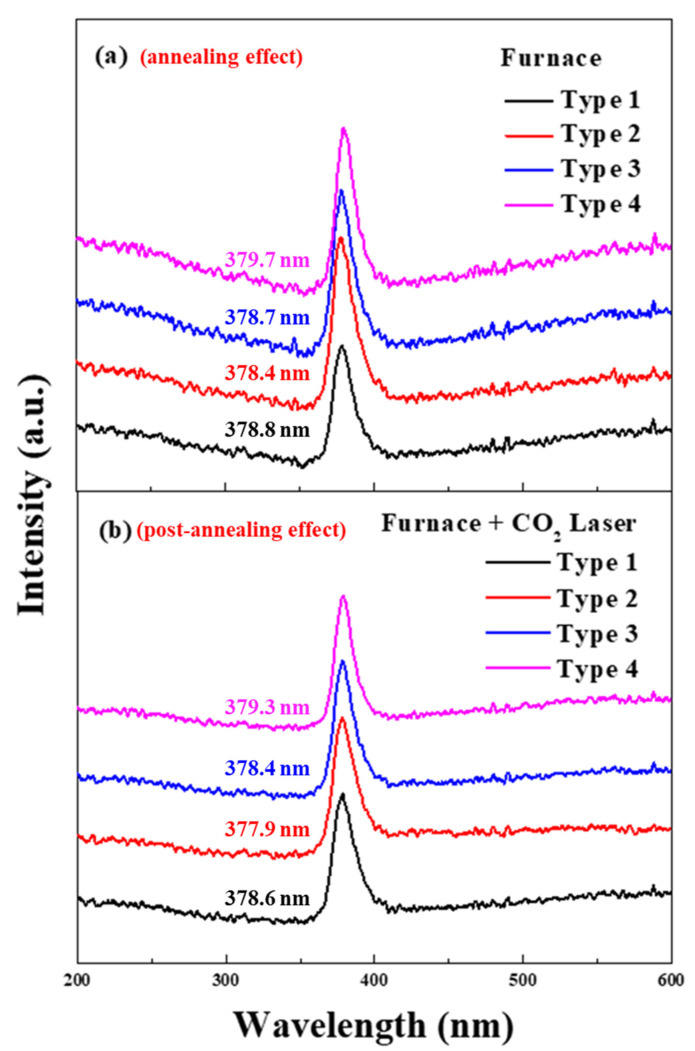
Photoluminescence spectra of 4 types of (In, Ga) co-doped ZnO multilayered thin films after electrical furnace-annealing effect (**a**) and furnace & CO_2_ laser annealing–post-annealing effect (**b**).

Figure 7 shows the energy band gaps of the four different types of (In, Ga) co-doped ZnO multi-deposition thin films. The calculated energy band gap was derived by employing Tauc’s plot. Figure 8a,b shows the energy band gaps of the thin films after the electrical furnace and after the furnace and carbon-dioxide laser annealing, respectively. By comparing Figure 8a,b, we can determine that the energy band gap slightly rose after carbon-dioxide laser annealing was performed. This increased energy band gap originates from the Burstein–Moss effect [41,42]. Carbon-dioxide laser annealing induced an increase in carrier concentration. This results in a shift of the Fermi level into the conduction band, which leads to the broadening of the optical bandgap energy. Moreover, we believe that the slight increase in the energy bandgap is caused by the elimination of defects such as point defect in the thin film by CO_2_ laser heat treatment and the release of trapped electrons.

Figure 8 illustrates the photoluminescence (PL) spectra of the four different types of (In, Ga) co-doped multi-deposition thin films after electrical furnace (a) and after electrical furnace and carbon-dioxide laser post annealing processes. All types of (In, Ga) co-doped multi-deposition thin films depicted major luminescent peaks at approximately 380 nm. When the excited electron generated by the He-Ag laser returns to the ground state, light is emitted. The wavelength of the light became shorter as the energy band gap increases. The energy band gap can be enlarged by the increase of carrier concentration derived from the Burstein–Moss effect. Table 3. explains each type’s energy band gap calculated by the following equation [43]:

(10) $$E=\frac{hc}{\lambda }$$

where h is the Planck constant, c is the velocity of light, and λ is the wavelength of the excited photons detected by the luminescent peaks in the PL spectrum. The peak position slightly moved towards a shorter wavelength, with an increase in the optical energy band gap derived after the carbon-dioxide laser irradiation process. We believe this increased energy bandgap seems to be related to the blue-shift of the dopant ionization process of dopant. The energy level of the donor dopants (In, Ga and Ti) was positioned near and below the conduction energy band. Therefore, the energy required for the ionization process was sufficiently provided by the CO_2_ laser annealing. As a result, many ionizations occur during the post-heat treatment process, and the energy band gap increases due to the blue shift effect.

**Figure 9:**
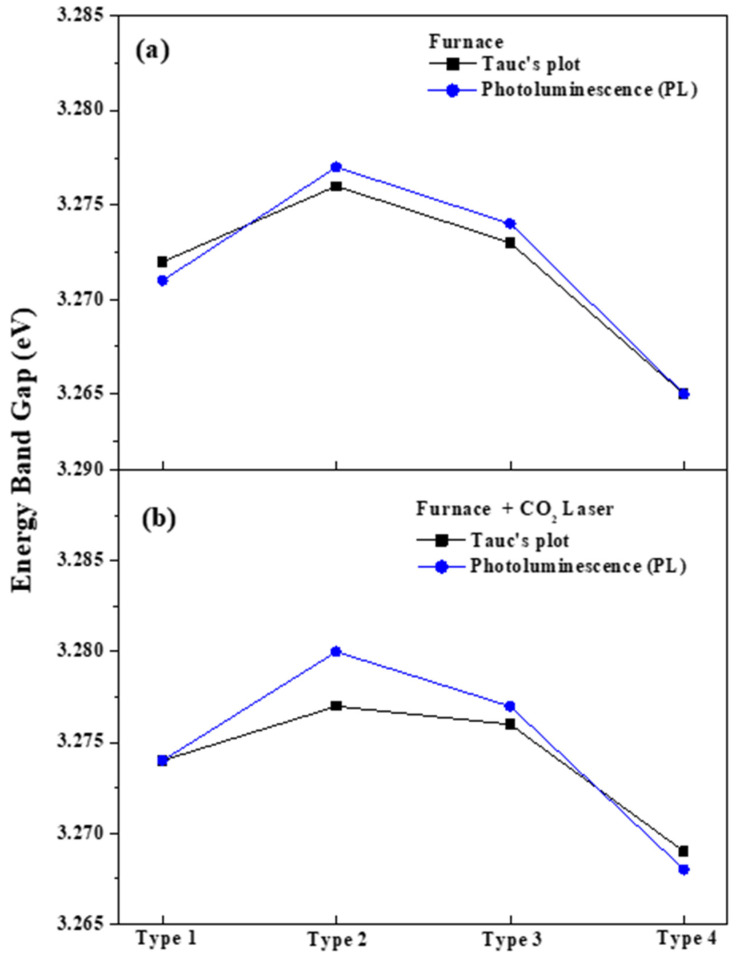
Energy band gap of 4 types of (In, Ga) co-doped multilayered thin films extracted from Tauc’s plot and measured by PL spectra.; (**a**) annealing effect, (**b**) post-annealing effect.

Figure 9 displays the energy band gap of the four types of (In, Ga) co-doped multi-deposition thin films extracted and derived by Tauc’s plot and PL spectra, respectively. We can see that the energy band gap data of Figure 9 and Table 3 have a similar tendency to Tauc’s plot presented in Figure 8. Thus, it can be confirmed that the electrical conductivities of the (In, Ga) co-doped ZnO multi-deposition samples were enhanced by carbon-dioxide laser annealing. As in the case reported in many studies, the energy is slightly increased when post-heat treatment is performed on the ZnO-based doped material. Therefore, we confirmed that the energy band gap increased due to the release in the number of electrons by defect removal. In addition, the blue-shifted effect was caused by the ionization reaction of the dopant by the CO_2_ laser post-annealing. Consequently, we believe that GZO (type 2) has the best characteristics when it is introduced as the bottom layer.

**Figure 10:**
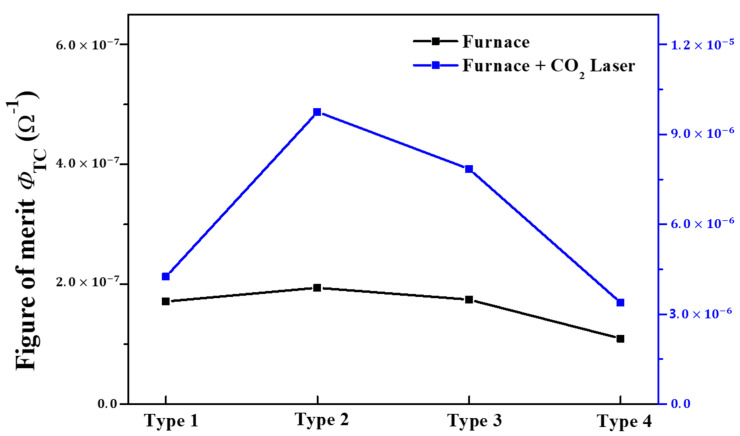
Figure of merit of four types of (In, Ga) co-doped multilayered thin films after electrical furnace and furnace & CO_2_ laser annealing.

Figure 10 illustrates the Figure of merit for four different types of (In, Ga) co-doped multi-deposition thin films defined by Haacke [44]:

(11) $$\phi_{TC}=\frac{T^{10}}{R_{s}}$$

where T is the optical transmittance value of the visible region in the wavelength range of 400 to 800 nm, and R_s is the sheet resistance of the thin films. For optical device applications, low sheet resistance and high optical transmittance are required. Therefore, Haacke’s figure of merit can be a good indicator of its ability for optical device applications. As shown in Figure 10, type 2 showed the highest figure of merit value of 9.75 × 10^−6^ among the four different types of (In, Ga) co-doped multi-deposition thin films. This result indicates that type 2 is the most suitable structure and process for optical applications. 

## 4. Conclusions

In this study, modeling of optimized lattice mismatch by carbon-dioxide laser annealing on (In, Ga) co-doped ZnO multi-deposition thin films was successfully investigated with crystallography and optical analysis. For the type 2 specimen, the Ga-doped ZnO thin film was prepared as the bottom layer, with the lowest lattice mismatch between the substrate and the thin film. This film showed the lowest resistance value of 34.5 kΩ/sq. among the specimens. We believe that the carbon-dioxide laser energy was used to relieve the stress in the microstructure of the thin film, and the remaining energy acted as a driving force for grain growth. However, the stress remaining in the thin film adversely affects grain growth. Type 2 exhibited the lowest residual stress in the thin film. Therefore, type 2 showed the largest grain size and grain growth value of 22.81 and 1.37 nm, respectively. In addition, type 2 formed an optimized structure in terms of lattice mismatch, and thus exhibited the lowest electrical conductivity and more than 85% of the optical transmittance in the visible region. Therefore, lattice mismatch control can enhance the carbon-dioxide laser annealing effect on thin films for optical device applications.

## Figures and Tables

**Table 1:** (002) peak angle, FWHM, lattice parameter c, and lattice parameter a for the ZnO-based thin films with 2 different annealing processes; F: electrical furnace annealing, FL: electrical furnace + carbon-dioxide laser annealing.

Materials	(002) Peak Angle (2 θ) (F/FL)	FWHM (F/FL)	Lattice Parameter c (Å) (F/FL)	Lattice Parameter A (Å) (F/FL)
IZO	34.5927/34.5723	0.44/0.41	5.1797/5.1827	3.2329/3.2348
GZO	34.4744/34.4458	0.51/0.22	5.1969/5.2011	3.2437/3.2463
IGZO	34.5112/34.4703	0.47/0.33	5.1915/5.1975	3.2403/3.2441
TZO	34.6213/34.5927	0.63/0.32	5.1755/5.1797	3.2303/3.2329

**Table 2:** Grain size and grain size difference of (In, Ga) co-doped multilayered thin films with the 2 different annealing processes; F: electrical furnace annealing, FL: electrical furnace + carbon-dioxide laser annealing.

Type	Crystallite Size (nm) (F)	Crystallite Size (nm) (FL)	Crystallite Size Difference (nm)
Type 1	21.16	21.82	0.66
Type 2	21.44	22.81	1.37
Type 3	21.32	22.09	0.77
Type 4	19.95	20.56	0.61

**Table 3:** Peak wavelength and energy band gap of (In, Ga) co-doped multilayered thin films with 2 different annealing processes; F: electrical furnace annealing, FL: electrical furnace + carbon-dioxide laser annealing.

Type	Peak Wavelength (nm) (F)	Peak Wavelength (nm) (FL)	Energy Band Gap (eV) (F)	Energy Band Gap (eV) (FL)
Type 1	378.8	378.6	3.271	3.274
Type 2	378.4	377.9	3.277	3.280
Type 3	378.7	378.4	3.274	3.277
Type 4	379.7	379.3	3.265	3.268

## Data Availability

The data presented in this study are available on request from the corresponding author.
